# Oral Excretion Kinetics of Food-Additive Silicon Dioxides and Their Effect on In Vivo Macrophage Activation

**DOI:** 10.3390/ijms25031614

**Published:** 2024-01-28

**Authors:** Ri-Ye Kwon, Su-Min Youn, Soo-Jin Choi

**Affiliations:** Division of Applied Food System, Major of Food Science & Technology, Seoul Women’s University, Seoul 01797, Republic of Korea; mystic2121@swu.ac.kr (R.-Y.K.); smyoun@swu.ac.kr (S.-M.Y.)

**Keywords:** silicon dioxide, food additives, oral excretion kinetics, fates, silicic acid, in vivo macrophage activation

## Abstract

A food additive, silicon dioxide (SiO_2_) is commonly used in the food industry as an anti-caking agent. The presence of nanoparticles (NPs) in commercial food-grade SiO_2_ has raised concerns regarding their potential toxicity related to nano size. While recent studies have demonstrated the oral absorption and tissue distribution of food-additive SiO_2_ particles, limited information is available about their excretion behaviors and potential impact on macrophage activation. In this study, the excretion kinetics of two differently manufactured (fumed and precipitated) SiO_2_ particles were evaluated following repeated oral administration to rats for 28 d. The excretion fate of their intact particles, decomposed forms, or ionic forms was investigated in feces and urine, respectively. Monocyte uptake, Kupffer cell activation, and cytokine release were assessed after the oral administration of SiO_2_ particles. Additionally, their intracellular fates were determined in Raw 264.7 cells. The results revealed that the majority of SiO_2_ particles were not absorbed but directly excreted via feces in intact particle forms. Only a small portion of SiO_2_ was eliminated via urine, predominantly in the form of bioconverted silicic acid and slightly decomposed ionic forms. SiO_2_ particles were mainly present in particle forms inside cells, followed by ionic and silicic acid forms, indicating their slow conversion into silicic acid after cellular uptake. No effects of the manufacturing method were observed on excretion and fates. Moreover, no in vivo monocyte uptake, Kupffer cell polarization, or cytokine release were induced by orally administered SiO_2_ particles. These finding contribute to understanding the oral toxicokinetics of food-additive SiO_2_ and provide valuable insights into its potential toxicity.

## 1. Introduction

Silicon dioxide (SiO_2_), a synthetic amorphous silica, is one of the most widely used food additives and plays a role as an anti-caking agent in processed foods, such as coffee creamer, seasoning, and powdered products. Registered as E551 in the European Union (EU), SiO_2_ has been assigned an acceptable daily intake (ADI) of ‘not specified’ by the Joint Food and Agriculture organization/Word Health Organization Expert Committee on Food Additives when employed as anti-caking agent [1,2]. The United State Food and Drug Administration (FDA) and Korea Food Additives Code recommend that SiO_2_ usage should not exceed 2% by weight of the food [3,4]. Depending on the manufacturing process, fumed and precipitated SiO_2_ are the most widely used directive additives in commercial foods [5,6]. Fumed (pyrogenic) SiO_2_ is produced through flame pyrolysis of silicon tetrachloride or by vaporizing quartz sand in an electric arc at 3000 °C [7,8]. Precipitated SiO_2_, also called as silicate hydrate, is generated through precipitation from a basic silicate solution with a mineral acid [9]. Despite having the same chemical formula, these two types of SiO_2_ particles exhibit differences in physicochemical properties, solubility, intestinal transport efficiency, and fate under various biological conditions [10,11,12]. The different characteristics of SiO_2_, arising from manufacturing methods, can also lead to unexpected biological responses and toxicity.

Our previous research reported the oral toxicokinetics of fumed and precipitated SiO_2_ particles after a single-dose administration to rats, showing ~3.1% and ~3.9% absorption for the former and the latter, respectively [13]. Tissue distribution patterns demonstrated that both SiO_2_ particles accumulated in only the liver at 28 d following repeated oral administration of 2000 mg/kg to rats for 28 d [13]. Notably, a significantly higher accumulation of precipitated SiO_2_ than fumed SiO_2_ was found in the liver, but the elevated levels returned to normal basal levels at 1 d of the recovery period [13]. Moreover, remarkably decomposed particles forms were only detected in the liver at 28 d after repeated oral administration of 2000 mg/kg SiO_2_ [13]. However, the excretion kinetics of SiO_2_ have not been clearly demonstrated yet, which can provide crucial information about tissue distribution kinetics, organ burden, and oral toxicokinetics.

An additional crucial question is whether SiO_2_ particles persist in intact particle forms or undergo decomposition into ionic forms or conversion into bioavailable forms following oral intake. SiO_2_, as a silicic acid anhydride of monomeric ortho-silicic acid (H_4_SiO_4_), is water-soluble and stable under aqueous conditions [14]. Indeed, ortho-silicic acid is known as the primary bioavailable form of silicon (Si) within the body [14,15]. It was reported that silicate, a group of polyatomic anions composed of silicon and oxygen in tetrahedral SiO_4_^4−^ units, undergoes decomposition into bioavailable ortho-silicic acid under acidic conditions, such as gastric juice, and is absorbed into the body in this form [14,15,16]. To date, clear information regarding the excretion fate of SiO_2_ following oral intake and intracellular fates after cellular internalization remains unavailable.

In general, food-additive SiO_2_ is considered as safe at the actual usage levels in processed foods [5]. Nevertheless, recent studies have demonstrated the potential toxicity of SiO_2_ in terms of inflammation, intestinal function, and neurotoxicity, although these studies were conducted in cultured cell lines [17,18,19]. Notably, the effects of SiO_2_ on inflammation responses and immune systems have been predominantly demonstrated through inhalation exposure, rather than oral intake [20,21]. Inflammation responses are intricately linked to macrophage activation, involving M1 and M2 macrophage polarization [22]. M1 macrophages are polarized by external stimuli such as lipopolysaccharide (LPS) and interferon-γ (INF-γ) and express CD80 and CD86 surface proteins [23]. M1 macrophage regulates the production of pro-inflammatory cytokines, including tumor necrosis factor-α (TNF-α), and interleukin (IL)-1β, IL-6, and IL-8, finally leading to inflammatory responses [24,25,26]. On the other hand, M2 macrophages are activated in response to certain cytokines, such as IL-4, IL-10, or IL-13, and identified by surface phenotypic markers including arginase-1, CD163, and CD206 [27,28,29]. The activation of an M2 macrophage contributes to wound healing, anti-inflammatory responses, and tissue repair [22,28,30]. Importantly, macrophage activation is dynamically regulated by cell signaling pathways, allowing for the coexistence of M1 and M2 macrophages with a mixed phenotype [31,32]. Indeed, transitions between M1 and M2 macrophage phenotypes were reported, with M1 macrophage converting into M2 or M2 macrophage being re-polarized to M1, although the mechanisms underlying these transitions remain not fully understood [31,33].

In this study, we evaluated the excretion kinetics of food-grade fumed and precipitated SiO_2_ particles following repeated oral administration to rats for 28 d, followed by a recovery period of 90 d. Their excretion fates in feces and urine were determined by transmission electron microscopy (TEM), molybdenum blue assay, and inductively coupled-atomic emission spectroscopy (ICP-AES). Macrophage uptake, macrophage activation, and cytokine release were also investigated after the repeated oral administration of two differently manufactured SiO_2_ particles for 28 d. Furthermore, the study explored intracellular particle or ionic fates and the conversion of bioavailable silicic acid inside macrophage Raw 264.7 cells.

## 2. Results and Discussion

### 2.1. Characterization and Quantitative Analytical Validation of SiO_2_

The constituent particle size, size distribution, and shape of fumed and precipitated SiO_2_ particles were examined by TEM, showing average particle sizes of 13.9 ± 3.2 nm and 16.8 ± 4.1 nm for the former and the latter, respectively (Figure S1). No significant differences were found in particle size between two particles (*p* > 0.05), and round shapes and aggregate formation were also examined.

**Figure 1:**
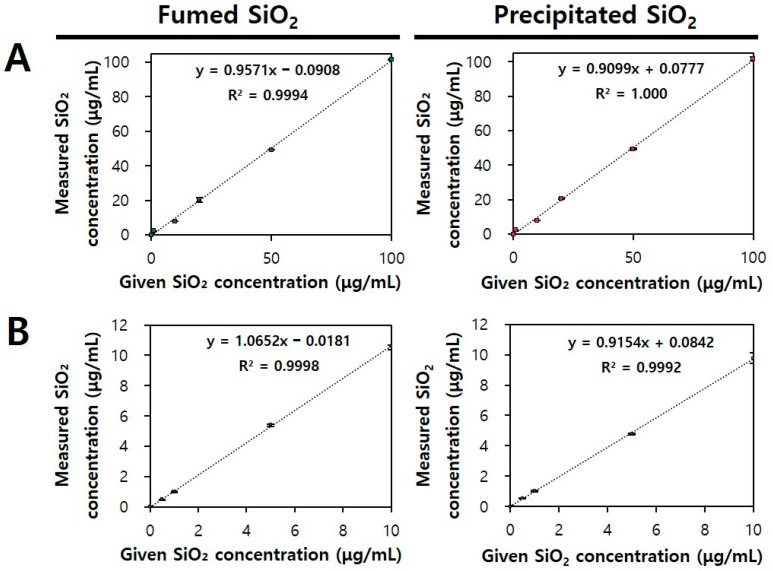
Standard curves for fumed SiO_2_ (green dots) and precipitated SiO_2_ (red dots) in (**A**) feces and (**B**) urine by microwave digestion and inductively coupled plasma atomic emission spectroscopy (ICP-AES) analysis.

To analyze the excretion kinetics of SiO_2_ following oral administration, a quantitative analytical method for SiO_2_-spiked feces and urine was validated. A microwave digestion system with nitric acid (HNO_3_) and hydrofluoric acid (HF) was applied to decompose organic matrices, and ICP-AES analysis was performed to quantify total Si amounts. Figure 1 demonstrates that both fumed and precipitated SiO_2_ spiked with the matrices had good linearity (R^2^). Table 1 shows that the analytical recovery (%) of SiO_2_ particles from both feces and urine ranged from 91.05% to 106.39%, suggesting the reliability of the method used. Small values of the coefficient of variation (CV) and relative error (RE) were also obtained, indicating the precision (CV) and accuracy (RE) of the method analyzed. The limit of detection (LOD) values of SiO_2_ were 165.24–185.98 μg/g feces and 24.84–38.17 μg/g urine, and the limit of quantification (LOQ) values were 500.72–563.58 μg/g feces and 75.28–115.67 μg/g urine, respectively. These LOQ values in urine were much lower than the values obtained by Dekkers et al. (642 μg/g foods) and by Yu et al. (329–416 μg/g foods), but the LOQ values in feces were similar or slightly higher than the values obtained in previous reports [34,35]. The slightly higher LOQ values in feces compared to those in urine seem to be strongly associated with a high matrix effect of rat diet composition. All analytical parameters indicate the reliability of the analytical methods used in this study.

### 2.2. Fecal Excretion Kinetics of SiO_2_

**Figure 2:**
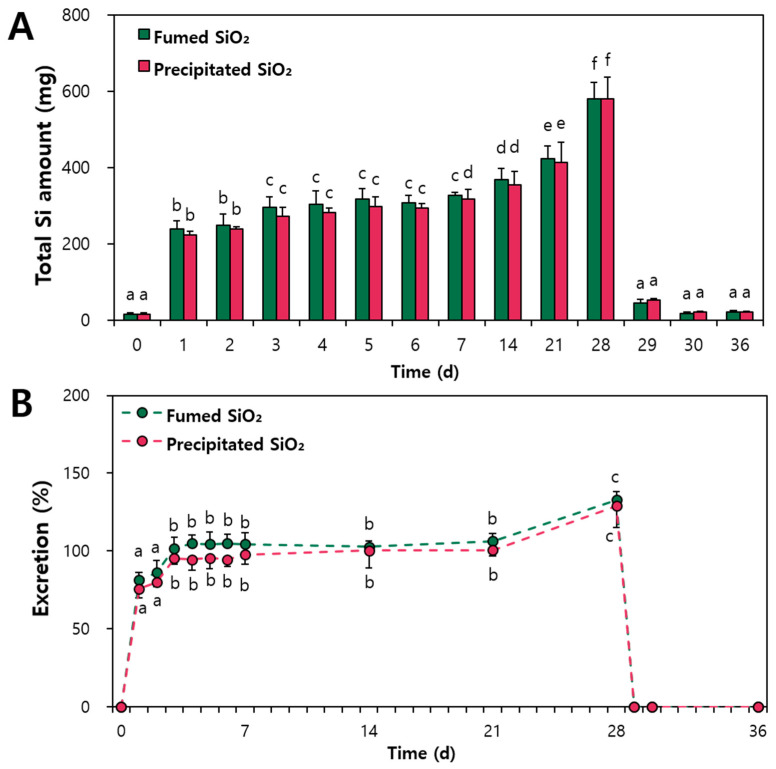
(**A**) Fecal excretion kinetics of fumed SiO_2_ and precipitated SiO_2_ and (**B**) their fecal excretion percentages (%) based on administered dose (2000 mg/kg) following repeated oral administration to rats for 28 d (number of animals = 6). Different lowercase letters (a–f) within a row in the same SiO_2_ indicate significant differences in SiO_2_ levels among different time points (*p* < 0.05). No significant differences were found between fumed SiO_2_ and precipitated SiO_2_ (*p* > 0.05).

The excretion kinetics of SiO_2_ was investigated following repeated oral administration of 2000 mg/kg for 28 d. A high dose was administered, the level of SiO_2_ at which there was no observed adverse effect was 2000 mg/kg [5,13], and low urinary excretion levels were expected. Figure 2A shows that the amounts of fumed and precipitated SiO_2_ particles excreted via feces significantly increased during the treatment period in a time-dependent manner and reached the highest level at 28 d, the final day of repeated administration. On the first day of the recovery period after 29 d, the increased levels returned to the normal basal level without significant differences compared to the non-treated control. The same tendency was found when the excretion percentages (%) via feces were calculated based on administered doses, showing ~80%, ~100%, and ~130% excretion at 1–2 d, 3–21 d, and 28 d, respectively (Figure 2B). These results suggest that most orally administered SiO_2_ particles were not absorbed but directly eliminated from the body via feces, which is consistent with their low oral absorption (~3.1–3.9%) demonstrated in our previous report [13]. The elevated excretion percentage observed at 28 d (~130%) seems to be associated with delayed fecal excretion following the repeated administration of a high dose (2000 mg/kg). However, it is worth noting that elevated total Si levels returned to normal at 1 d of recovery period, just after stopping administration, implying that food-grade SiO_2_ is safe at actual usage levels. On the other hand, no statistical significances were found between fumed SiO_2_ and precipitated SiO_2_, probably related to their large amount of excretion and low oral absorption levels (~3.1% and ~3.9% for the former and the latter, respectively) [13].

### 2.3. Urinary Excretion Kinetics of SiO_2_

**Figure 3:**
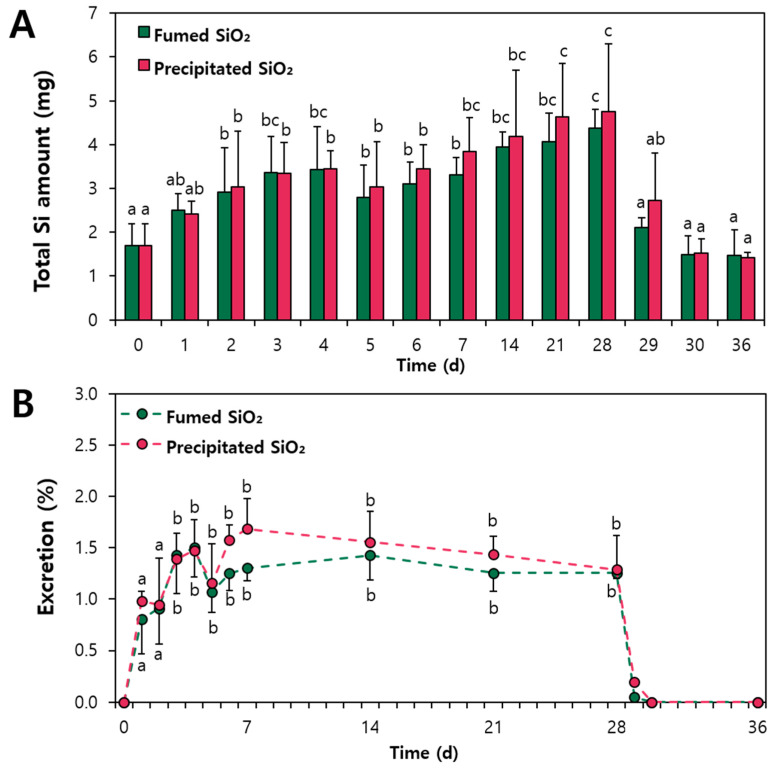
(**A**) Urinary excretion kinetics of fumed SiO_2_ and precipitated SiO_2_ and (**B**) their urinary excretion percentages (%) based on administered dose (2000 mg/kg) following repeated oral administration to rats for 28 d (number of animals = 6). Different lowercase letters (a–c) within a row in the same SiO_2_ indicate significant differences in SiO_2_ levels among different time points (*p* < 0.05). No significant differences between fumed SiO_2_ and precipitated SiO_2_ were found (*p* > 0.05).

The oral urinary excretion kinetics of SiO_2_ (2000 mg/kg) demonstrated that the amounts of fumed SiO_2_ and precipitated SiO_2_ via urine also increased in a time-dependent manner during the administration period (Figure 3A). However, no significant elevated total Si levels compared to the non-treated control were found at 29 d, the first day of the recovery period, suggesting that orally absorbed SiO_2_ can be eliminated by the kidney within 24 h. This result also supports the safety aspect of food-additive SiO_2_ at actual usage levels. No statistical differences were found between two SiO_2_ particles, which can be explained by the small difference in oral absorption (~3.1% and ~3.9% for fumed and precipitated SiO_2_, respectively) [13]. The excretion percentages (%) of SiO_2_ via urine ranged from ~1% to ~2% (Figure 3B), suggesting that most absorbed SiO_2_ particles could be eliminated by urinary excretion. The differences between absorption and urinary excretion amounts are likely to be related to the low remaining levels of SiO_2_ in the metabolic excretion cage despite the washing process. It was reported that SiO_2_ can be excreted by the kidneys in proportion to the amount absorbed [13,36,37]. On the other hand, elevated total Si levels were detected at 0 d and 29 d (Figure 3A), before and after administration, respectively, which is surely related to high contents of Si in animal feeds [38,39]. Taken together, all the excretion kinetic results suggest that most orally administered SiO_2_ particles were directly excreted via feces and that absorbed particles are primarily eliminated from the body by the kidneys.

### 2.4. Fecal Excretion Fates of SiO_2_

**Figure 4:**
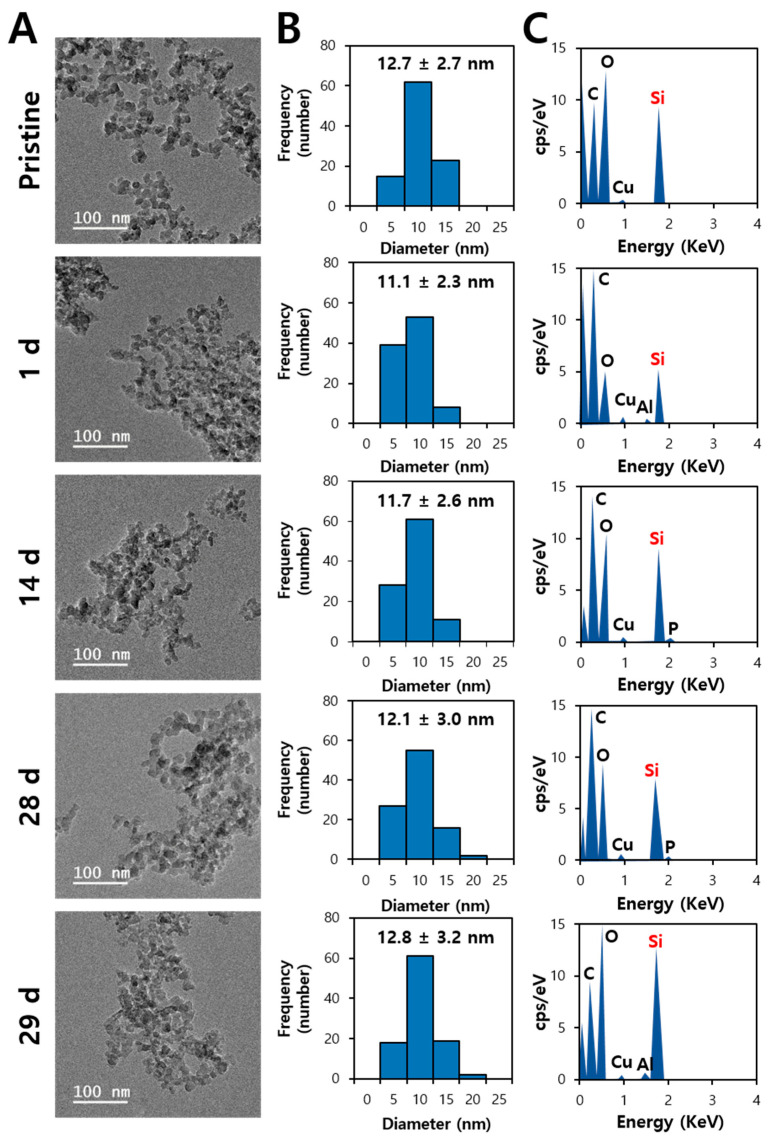
(**A**) Transmission electron microscopy (TEM) images, (**B**) particle size distribution, and (**C**) energy-dispersive X-ray spectroscopy (EDS) of pristine SiO_2_ and fumed SiO_2_ excreted via feces following repeated oral administration (2000 mg/kg) to rats for 28 d (number of animals = 6). No significant differences in particle size were found between fumed SiO_2_ and precipitated SiO_2_ (*p* > 0.05).

**Figure 5:**
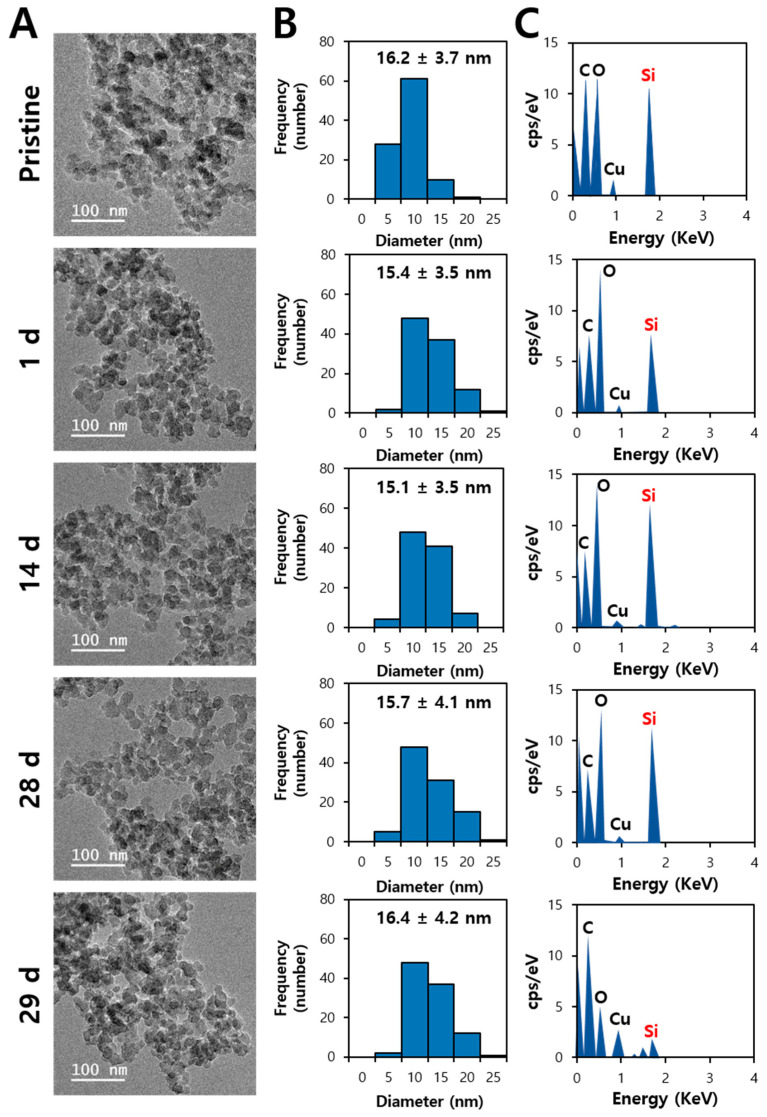
(**A**) Transmission electron microscopy (TEM) images, (**B**) particle size distribution, and (**C**) energy-dispersive X-ray spectroscopy (EDS) of pristine SiO_2_ and precipitated SiO_2_ via feces following repeated oral administration (2000 mg/kg) to rats for 28 d (number of animals = 6). No significant differences were found in particle size between fumed SiO_2_ and precipitated SiO_2_ (*p* > 0.05).

TEM–energy-dispersive X-ray spectroscopy (EDS) analysis was performed to answer the question as to whether orally administered SiO_2_ was excreted via feces in decomposed small molecules or intact particle forms. Mild acid digestion was carried out with 60% HNO_3_ and 34.5% hydrogen peroxide (H_2_O_2_) for excreted feces. This did not dissolve SiO_2_ particles into Si ions, but only digest organic matrices [35]. Figure 4A and Figure 5A demonstrate that both fumed and precipitated SiO_2_ particles in feces remained in particle forms over a period of administration. When the particle size distribution was measured from the TEM images, the average particle sizes of fecal excreted SiO_2_ over the treatment period were statistically the same as those of pristine SiO_2_ before administration (Figure 4B and Figure 5B). TEM-EDS analysis also revealed the presence of Si in the fecal excreted particles (Figure 4C and Figure 5C). No significant difference in constituent particle size was found between pristine fumed SiO_2_ and precipitated SiO_2_ before administration, which is in good agreement with the TEM results (Figure S1) and our previous report [10]. All the results suggest that orally administered SiO_2_ particles can be excreted via feces in intact particle forms without decomposition.

### 2.5. Urinary Excretion Fates of SiO_2_

**Figure 6:**
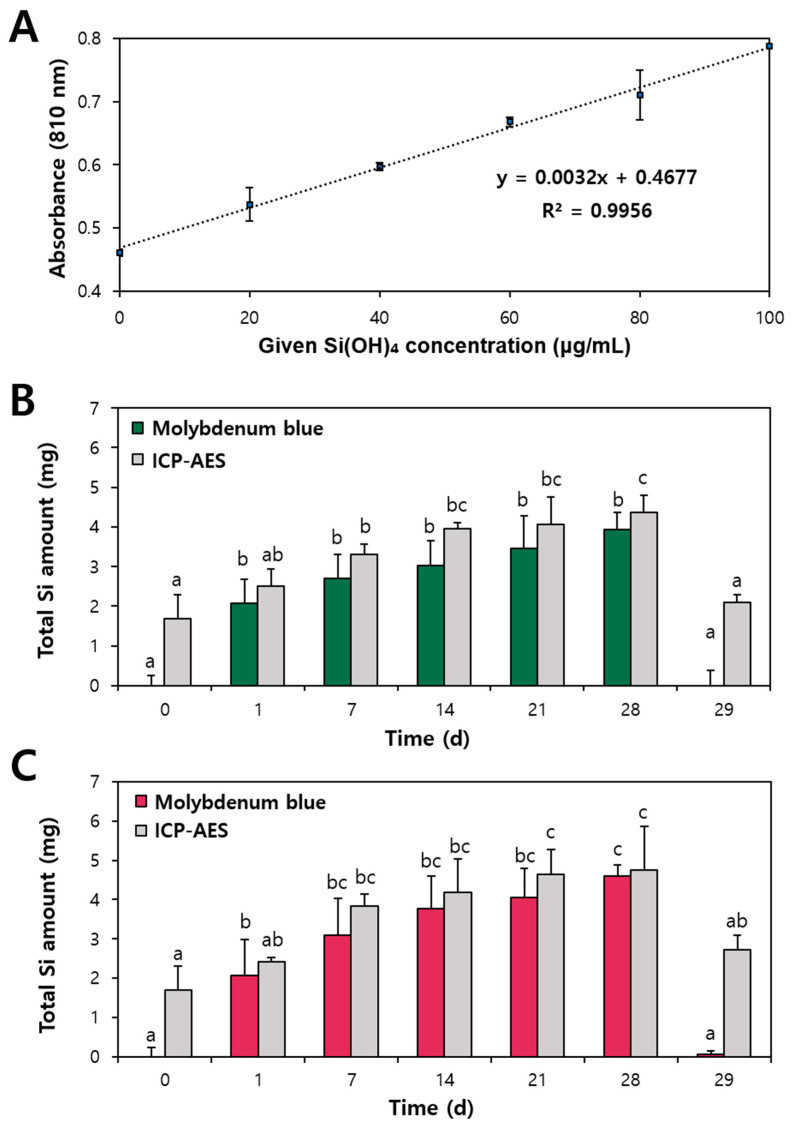
(**A**) Standard curve of silicic acid assessed by a molybdenum blue assay in urine (number of repetitions = 5) and comparison of total Si amounts measured by molybdenum blue method and by inductively coupled plasma atomic emission spectroscopy (ICP-AES) following repeated oral administration (2000 mg/kg) of (**B**) fumed SiO_2_ and (**C**) precipitated SiO_2_ to rats for 28 d (number of animals = 6). Different lowercase letters (a–c) indicate significant differences among different time point (*p* < 0.05).

The urinary excretion fate of SiO_2_ following oral administration was evaluated using the molybdenum blue method. Indeed, urinary excretion of SiO_2_ was reported in several studies by quantifying total Si levels in urine [5,36,40]. The water-soluble and bioavailable form of SiO_2_ is known to be a silicic acid anhydride of monomeric ortho-silicic acid [14,41], and the ortho-silicic acid can be determined via molybdenum blue complex analysis [42,43]. This is based on the reaction of soluble SiO_2_ with an excess of ammonium molybdate at pH 1.5–2, resulting in silicomolybdate complex formation. The molybdenum blue method is a spectrophotometric assay, which is a simple and cost-effective technique used to determine silicic acid levels [44]. It is worth noting that the molybdenum blue method has the advantage of determining the soluble ortho-silicic acid form of SiO_2_, whereas ICP-AES only analyzes the total Si amounts [43]. Quantitative analysis of SiO_2_ conversion into silicic acid after oral intake via excreted urine has not been clearly shown. Figure 6A demonstrates the linearity of the molybdenum blue method used for quantitative analysis of ortho-silicic acid in urine.

Determined amounts of ortho-silicic acid in urine were converted into total Si levels and a comparison between molybdenum blue method and ICP-AES analysis was also carried out. As shown in Figure 6B,C, about 76.5–96.8% of total Si levels in urine detected by ICP-AES were determined to be in an ortho-silicic acid form at 1–28 d of administration, suggesting that most SiO_2_ excreted via urine was in an ortho-silicic acid form. These results are consistent with previous reports [14,45,46] suggesting the bioconversion of SiO_2_ into soluble ortho-silicic acid after oral administration. Hence, orally absorbed SiO_2_ can be primarily excreted in silicic acid via urine. On the other hand, no significantly elevated Si levels were detected at 0 d and 29 d by the molybdenum blue method despite the increased total Si amounts measured by ICP-AES. This result implies that total Si levels detected at 0 d and 29 d are only Si ions resulting from animal feeds, but not ortho-silicic acid forms from SiO_2_ particles. Typically, Si content in rodent diets ranges from 300 to 600 µg/g [47,48], with the rats in this study consuming a daily feed intake 20–25 g. Consequently, the estimated daily Si intake from the diets is in the range of 5 to 15 mg, which may explain the elevated Si levels in urine at 0 d.

### 2.6. In Vivo Monocyte Uptake of SiO_2_

**Figure 7:**
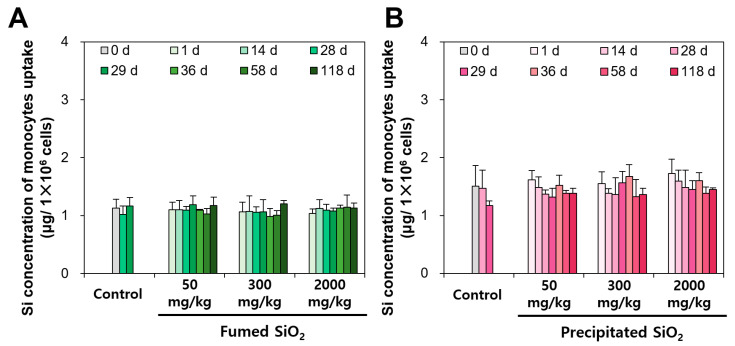
Total Si uptake concentrations in monocytes after 28 d repeated oral administration (2000 mg/kg) of (**A**) fumed SiO_2_ and (**B**) precipitated SiO_2_, followed by a recovery period of 90 d (number of animals = 5). No significant differences among control and SiO_2_ treated group were found (*p* > 0.05).

To evaluate potential macrophage activation caused by fumed and precipitated SiO_2_ particles, in vivo monocyte uptake was assessed by ICP-AES analysis following repeated oral administration for 28 d. A monocyte is a type of white blood cell (leukocyte) that can differentiate into macrophages and monocyte-derived dendritic cells, thereby playing a role in innate immune systems [49,50]. The results showed that no significant increase in monocyte uptake of SiO_2_ was found during the administration and recovery period up to high dose of 2000 mg/kg, regardless of manufactured type (Figure 7), implying the low potential of monocyte activation by orally administered food-additive SiO_2_ particles.

### 2.7. In Vivo Kupffer Cell Activation by SiO_2_

**Figure 8:**
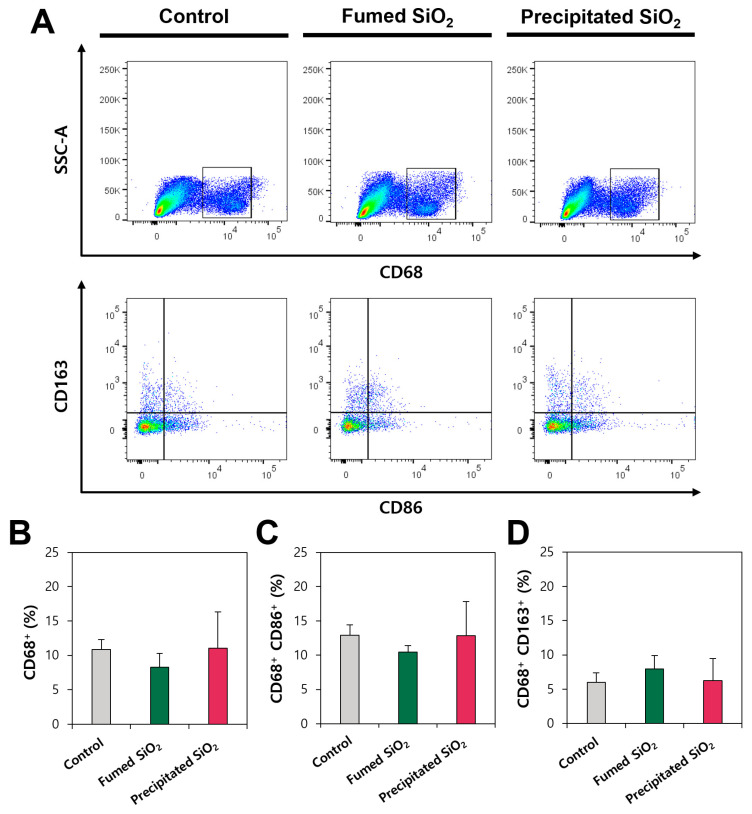
(**A**) Kupffer cell differentiation after repeated oral administration (2000 mg/kg) of SiO_2_ for 28 d by flow cytometry analysis. The percentages of (**B**) CD68 (pan-macrophage marker)-, (**C**) CD86 (M1 marker)-, and (**D**) CD163 (M2 marker)-positive cells (number of animals = 5). No significant differences among non-treated control, fumed SiO_2_-, and precipitated SiO_2_-treated groups were found (*p* > 0.05).

Kupffer cells are resident in the liver and comprise the largest population of hepatic macrophages, playing a role in the innate immune responses [51,52]. They remove foreign materials and particles taken up by the hepatic portal system through phagocytosis and pinocytosis [53]. The Kupffer cells are polarized into M1 and M2 phenotypes, secreting pro-inflammatory cytokines (IL-1β, IL-6, IL-8, IL-12, IL-23 and TNF-α) and anti-inflammatory cytokines (IL-10 and TGF-β1), respectively [52,54]. Since fumed and precipitated SiO_2_ particles were determined to accumulate in the liver in our previous report [13], the Kupffer cells were isolated from the liver and their activation was analyzed by flow cytometry following oral administration for 28 d. The results showed that no significant macrophage differentiation in the liver was found between non-treated control and fumed or precipitated SiO_2_-treated groups, as analyzed by a rat pan-macrophage marker CD68, M1 macrophage marker CD86, and M2 macrophage marker CD163, respectively (Figure 8A). When expression levels of CD68, CD86, and CD163 were compared, no statistical differences among control, fumed SiO_2_-, and precipitated SiO_2_-treated groups were found (Figure 8B–D) (*p* > 0.05), suggesting that M1 and M2 polarization in the Kupffer cells did not occur following the repeated oral administration of fumed or precipitated SiO_2_ for 28 d. These results also imply that the Kupffer cells were not activated despite the slight accumulation of SiO_2_ particles in the liver [13].

### 2.8. In Vivo Cytokine Release by SiO_2_

**Figure 9:**
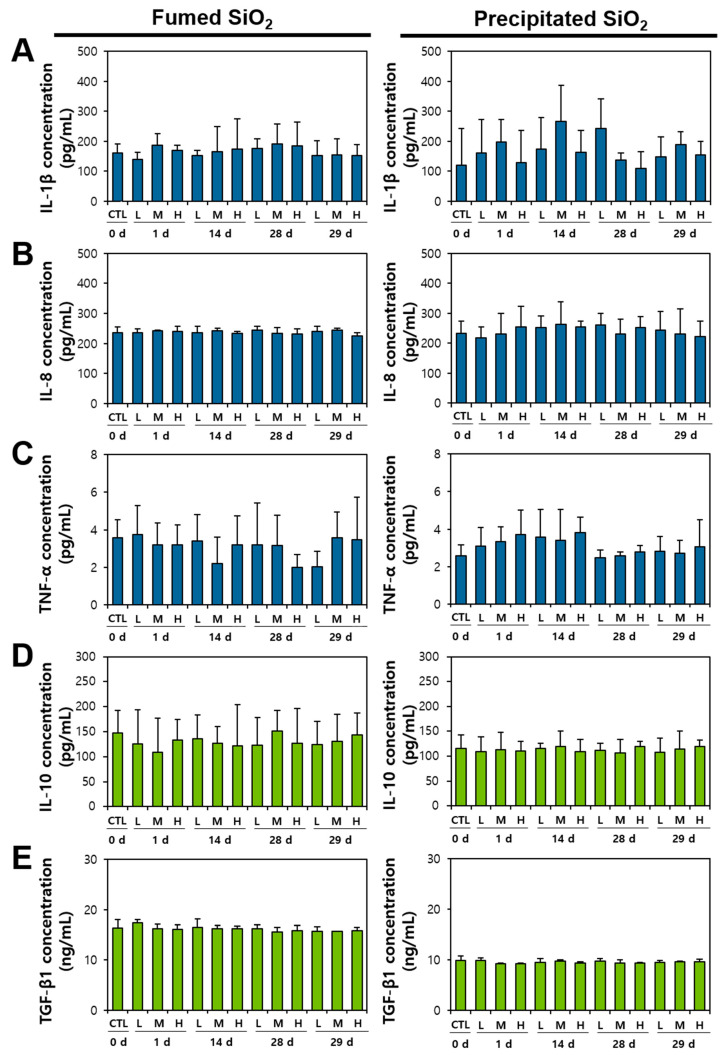
Induction of pro-inflammatory cytokines (**A**) IL-1β, (**B**) IL-8, (**C**) TNF-α and anti-inflammatory cytokines (**D**) IL-10 and (**E**) TGF-β1 into plasma after repeated oral administration (2000 mg/kg) of fumed SiO_2_ and precipitated SiO_2_ for 28 d, followed by a recovery period (number of animals = 5). No significant differences were found among non-treated control, fumed SiO_2_- and precipitated SiO_2_-treated groups, among different time points and among different doses (*p* > 0.05). Abbreviation: CTL, non-treated control; L, 50 mg/kg; M, 300 mg/kg; H, 2000 mg/kg.

The release of pro-inflammatory cytokines, such as IL-1β, IL-8, and TNF-α, and anti-inflammatory cytokines, such as IL-10 and TGF-β1, caused by M1 and M2 macrophage activation, respectively, was evaluated following oral administration for 28 d, although in vivo monocyte uptake (Figure 7) or M1 an M2 polarization (Figure 8) were not detected. Three different doses (50, 300, and 2000 mg/kg) were administered as cytokines have the potential to be released at even low dosage levels [55,56]. As mentioned in the previous section, the Kupffer cell polarization to M1 and M2 macrophages leads to the secretion of pro-inflammatory and anti-inflammatory cytokines, respectively [52,54]. Hence, cytokine release can be an indicator for the potential toxicity of inflammation or protection of inflammation response [57,58]. Figure 9 demonstrates that neither pro-inflammatory nor anti-inflammatory cytokines were secreted during and after the oral administration of fumed or precipitated SiO_2_ for 28 d. Taken together, it is strongly likely that orally taken SiO_2_ particles do not cause macrophage activation or pro- and anti-inflammatory cytokine release, regardless of manufactured type. These results seem to be attributed to their low oral absorption (~3–4%) and low accumulation in the liver [13], suggesting their safety as food additives.

### 2.9. In Vitro Cellular Uptake and Intracellular Fates of SiO_2_

**Figure 10:**
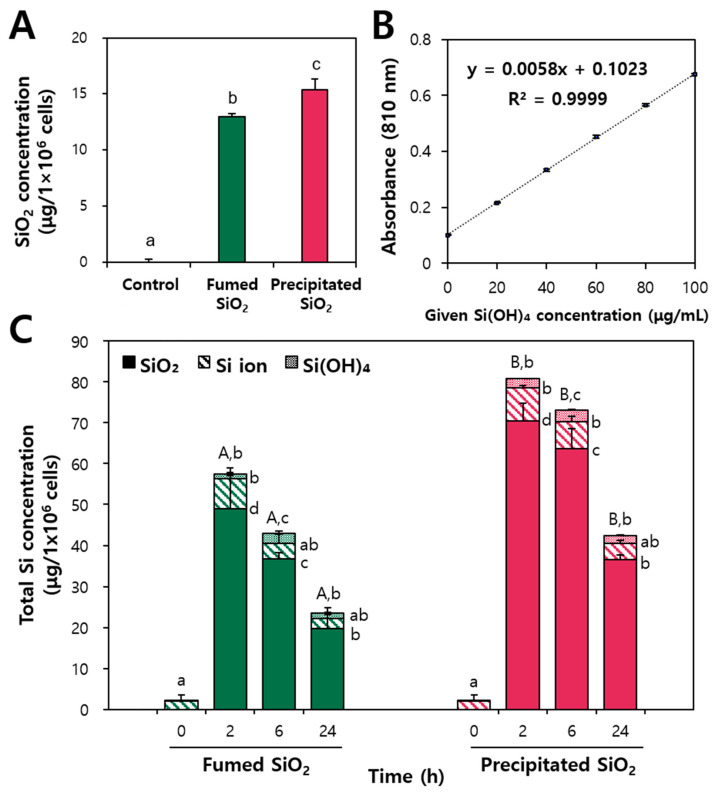
(**A**) Cellular uptake of fumed SiO_2_ and precipitated SiO_2_ in Raw 264.7 cells; (**B**) standard curve of cell suspension spiked with ortho-silicic acid (number of repetitions = 5); and (**C**) intracellular fates of fumed SiO_2_ and precipitated SiO_2_ after exposure for 24 h. Different uppercase letters (A,B) indicate significant differences between fumed SiO_2_ and precipitated SiO_2_ (*p* < 0.05). Different lowercase letters (a–d) indicate significant differences among control, fumed SiO_2_-, and precipitated SiO_2_-treated groups (*p* < 0.05).

The cellular uptake of fumed and precipitated SiO_2_ particles was also investigated by quantifying total cellular Si levels using ICP-AES after exposure for 24 h. Macrophage Raw 264.7 cells were selected due to their capacity to engulf and digest foreign substances, making them optimal for evaluating the intracellular fate of SiO_2_. The result showed that a significantly larger amount of precipitated SiO_2_ than fumed SiO_2_ was taken up by Raw 264.7 cells (Figure 10A), which must surely be related to the smaller hydrodynamic diameters of the former (156 ± 1 nm) than the latter (375 ± 1 nm) as demonstrated in our previous report [10]. Indeed, many studies demonstrated that small-sized particles can be more easily and massively internalized into cells than larger-sized particles [59,60,61].

The intracellular fates of SiO_2_ were evaluated to answer the question as to whether the particles were present in intact particle, dissolved ionic, or bioavailable ortho-silicic acid forms after cellular uptake. The particle and soluble (ionic and ortho-silicic acid) forms of SiO_2_ particles inside cells were separated via the cloud point extraction (CPE) approach, as demonstrated in our previous study, which can encapsulate particles in a surfactant-based micelle structure, thereby separating particles from ions [10]. Ortho-silicic acid and dissolved ionic forms were determined using the molybdenum blue assay and ICP-AES analysis, respectively. The standard curve of cell suspensions spiked with ortho-silicic acid showed a good linearity (Figure 10B). Figure 10C shows that the cells had major Si ions (~97.2%, ~2 μg/1 × 10^6^ cells) and a small portion of ortho-silicic acid (~2.8%, 0.06 μg/1 × 10^6^ cells) before SiO_2_ treatment, although total Si levels were extremely low. This result indicates that basal ionic and bioavailable Si forms were present inside cells. On the other hand, total absolute amounts of intact particles, ionic, and ortho-silicic acid forms were significantly higher in precipitated SiO_2_-treated cells than in fumed SiO_2_-treated ones, which is consistent with the uptake result (Figure 10A). When the fates of SiO_2_ were calculated by percentages, ~83.9–87.3%, ~8.9–12.9%, and ~2.1–5.7% were present in particles, Si ions, and ortho-silicic acid, respectively, after incubation for 2–24 h, without significant differences between fumed and precipitated SiO_2_ (*p* > 0.05). These results imply that SiO_2_ is primarily present in particles, but that a portion (~15%) of SiO_2_ can dissolve into Si ions and convert into ortho-silicic acid inside cells. Interestingly, the levels of silicic acid increased from ~2.3% (2 h) to ~5.0% (24 h) as incubation time increased. This result suggests that SiO_2_ particles can dissolve into Si ions, but also transfer to bioavailable ortho-silicic acid after cellular uptake. Acidic lysosomal condition inside cells may contribute to decomposing SiO_2_ and its conversion into bioavailable ortho-silicic acid form.

## 3. Materials and Methods

### 3.1. Materials

Food-grade fumed SiO_2_ (AEROSIL^®^ 200F) and precipitated SiO_2_ (SIPERNAT^®^ 22S) were purchased from Evonik Industries AG (Essen, NRW, Germany). For oral administration, SiO_2_ was dispersed in distilled water (DW) at predetermined concentrations (5, 30, or 200 mg/mL) and stirred for 30 min, followed by sonication for 15 min using a bath sonicator (160 Watts, Bransonic 5800, Branson Ultrasonics, Danbury, CT, USA). For in vitro cell experiments, SiO_2_ (1000 μg/mL) was suspended in cell culture medium and stirred for 30 min. All stock solutions were freshly prepared prior to treatment.

Si standard solution and TritonX-114 (TX-114) and collagenase IV were provided by Sigma-Aldrich (St. Louis, MO, USA). Sodium ortho-silicate was supplied by Alfa-Aesar (Haverhill, MA, USA). HNO_3_, H_2_O_2_, HF, sodium chloride (NaCl), hydrogen chloride (HCl), sodium hydroxide (NaOH), sulfuric acid (H_2_SO_4_), ammonium molybdate, oxalic acid dehydrate, ascorbic acid, and ethyl alcohol were purchased from Samchun Pure Chemical Co., Ltd. (Pyeongtaek, Gyeonggi-do, Republic of Korea). Ficoll-Paque™ PLUS was supplied by GE Healthcare (Chicago, IL, USA) and Percoll™ was obtained from Cytiva (Marlborough, MA, USA). Roswell Park Memorial Institute (RPMI) 1640, inactivated fetal bovine serum (FBS), penicillin, streptomycin, phosphate-buffered saline (PBS), Dulbecco’s phosphate-buffered saline (DPBS), and Hanks’ Balanced Salt Solution (HBSS) were purchased from Welgene Inc. (Gyeongsan-si, Gyeongsangbuk-do, Republic of Korea). Enzyme-linked immtunosorbent assay (ELISA) kits for rat IL-1β were supplied by Abcam (Cambridge, UK). ELISA kits for rat IL-8 were purchased from MyBioSource (San Diego, CA, USA), and rat TNF-α, rat IL-10, and rat TGF-β1 ELISA kits were obtained from Thermo Fisher Scientific (Waltham, MA, USA). Phycoerythrin (PE)-conjugated anti-rat CD86, Alexa Fluor 647-conjugated anti-rat CD163, and fluorescein isothiocyanate (FITC)-conjugated anti-rat CD68 antibodies were provided by Bio-Rad (Hercules, CA, USA). BD Cytofix/Cytoperm™ Fixation/Permeabilization kit and staining buffer were purchased from BD Biosciences (Franklin Lakes, NJ, USA).

### 3.2. Animals

Five-week-old female Sprague Dawley (SD) rats were purchased from Koatech Co. (Pyeongtaek, Gyeonggi-do, Republic of Korea), and excretion kinetics and Kupffer cell activation were evaluated and performed at Seoul Women’s University following repeated administration for 28 d and a recovery period of 90 d. The five-week-old female SD rats were supplied from G-Bio (Gwangju, Republic of Korea) for monocyte uptake following repeated administration for 28 d, followed by a recovery period of 90 d, which were performed at the Korea Testing & Research Institute (KTR, Gwacheon-si, Jeollanam-do, Republic of Korea). The rats were housed in a clean animal rack maintained at 20 ± 2 °C and 60 ± 10% relative humidity with a 12 h light–dark cycle. Water and the standard laboratory complete diet were given ad libitum. The animals were acclimatized for 7 d before experiments and used at six weeks old. All animal experiments were performed in accordance with the guide established by the Animal and Ethics Review Committee (IACUC) of Seoul Women’s University (SWU IACUC 2021-18) and KTR (IAC2020-2120; IAC2021-0417).

### 3.3. Excretion Kinetics following Repeated Oral Administration for 28 d

The six female SD rats were housed in metabolic cages and divided into two groups based on the manufacturing method of food-additive SiO_2_. The rats were repeatedly administered for 28 d via oral gavage at 2000 mg/kg to determine the excretion kinetics of SiO_2_. Urine and feces were collected before administration (0 d) and during the administration and recovery periods for 90 d. Feces were dried in a drying oven (HK-DO135F, Hankuk Scientific Instrument, Hwaseong, Gyeonggi-do, Republic of Korea) at 60 °C for 2 d and homogenized using an agate mortar. The remaining urine in the urine tubes was also collected by washing the tubes with 1 mL of distilled and deionized water (DDW), and centrifugation (2400× *g*, 25 °C) was carried for 5 min out to remove impurities. All samples were stored at −80 °C before analysis. Samples from the designated time points (0, 1, 2, 3, 4, 5, 6, 7, 14, 21, 28, 29, 30, and 36 d) were analyzed as described in Section 3.8. Excretion percentages (%) were determined at each time point by calculating the daily dose.

### 3.4. Excretion Fate Determination in Feces by TEM-EDS

The particle size and shape of excreted SiO_2_ particles before and after oral administration were measured using high-resolution TEM-EDS (JEM-2100F, JEOL, Tokyo, Japan). The suspension of pristine SiO_2_ (0.1 mg/mL) was prepared in ethyl alcohol and sonicated (160 W, Bransonic 5800, Branson Ultrasonics, Danbury, CT, USA) for 15 min. SiO_2_ particles in feces were separated after organic digestion at 190–200 °C using 60% HNO_3_ (10 mL) and 34.5% H_2_O_2_ (1 mL) until the solution was colorless and entirely evaporated, as previously described [35]. The particles were washed twice with DDW and dispersed in ethyl alcohol (0.1 mg/mL). After vortexing and sonication for 15 min, the pristine and feces-separated suspensions (5 µL) were loaded onto a carbon-coated copper grid (200 mesh, PELCO^®^ TEM Grids, Ted Pella Inc., Redding, CA, USA) and dried at room temperature. TEM images were acquired at an accelerating voltage of 200 kV. The particle sizes of SiO_2_ were determined using ImageJ software (version 1.53k, National Institutes of Health, Bethesda, MD, USA). Pristine SiO_2_ was processed before oral administration in the same method for comparison.

### 3.5. Excretion Fate Determination in Urine by Molybdenum Blue Assay

The ortho-silicic acid fate of SiO_2_ in urine was determined using a molybdenum blue assay [62]. Ammonium molybdate (3.1 g) dissolved in 50 mL of 1 M sulfuric acid. Oxalic acid dehydrate (6.3 g) and ascorbic acid (1.76 g) dissolved in 50 mL of DDW, respectively. Sodium ortho-silicate dissolved in DDW (1000 μg/mL, adjusted to pH 7 with HCl) was diluted with non-treated urine to designated concentrations (0, 20, 40, 60, 80, and 100 μg/mL) and used in ortho-silicic acid standard solutions. The urine (75 μL) was filtered through a 0.45 μm syringe filter, diluted 20-fold with DDW, and had its pH adjusted to 7. The urine solution was mixed with ammonium molybdate solution (75 μL) for 10 min. Then, oxalic acid solution (75 μL) was added and incubated for 1 min, followed by the addition of ascorbic acid solution (75 μL). After incubation for 10 min, absorbance was measured at 810 nm using a microplate reader (Infinite^®^ 200 PRO M Plex, Tecan, Männedorf, Switzerland).

### 3.6. Monocyte Uptake following Repeated Oral Administration for 28 d

Five female SD rats per group were administered three different doses (50, 300, and 2000 mg/kg) of fumed SiO_2_ and precipitated SiO_2_ via oral gavage for 28 d, followed by a recovery period of 90 d. At the designated time points (0, 1, 14, 28, 29, 36, 58, and 118 d), the rats were fasted for more than 18 h and anaesthetized with isoflurane solution. Their whole blood was then collected from the abdominal aorta. Blood monocytes were isolated using the Ficoll-Paque™ PLUS density gradient centrifugation method [63]. The blood (4 mL) was layered on 5 mL of Ficoll-Paque™ PLUS solution in 15 mL conical tube and centrifuged (400× *g*, 25 °C) for 40 min, and then the middle layer containing monocytes and lymphocytes was transferred to a new conical tube. PBS (13 mL) was added to the tube and centrifugation (200× *g*, 25 °C) was performed for 10 min to remove Ficoll-Paque™ PLUS solution. The cell pellets in the precipitates were re-suspended in 100 μL of PBS, followed by layering the suspension on 300 μL of Percoll™ solution. After centrifugation (580× *g*, 25 °C) for 15 min, the monocytes in the precipitates were collected and washed with 13 mL of PBS. After centrifugation (200× *g*, 25 °C) for 10 min, the monocytes in the precipitates were re-suspended in 200 μL of PBS. The cell viability of the isolated monocytes was more than 95.2%, as assessed by trypan blue staining. SiO_2_ uptake in the monocytes was quantified using ICP-AES as described in Section 3.8.

### 3.7. Kupffer Cell Activation following Repeated Oral Administration for 28 d

The livers of the rats administered 2000 mg/kg were collected at 29 d, which was 1 d of recovery period after euthanasia by CO_2_, and then washed twice with PBS. The hepatic macrophage Kupffer cells were isolated by density gradient centrifugation using Percoll™ solution [64,65]. The liver (1 g) was chopped with stainless-steel scissors, suspended in 50 mL of RPMI 1640 medium containing 0.05% collagenase IV, 0.002% DNase I, and 20% FBS, and incubated for 50 min at 37 °C with shaking. The digested tissue was then filtered through a cell strainer (100 μm) to remove undigested tissue, and centrifuged (250× *g*, 4 °C) for 10 min. The cell pellets in the precipitates were re-suspended in 50 mL of HBSS, followed by centrifugation (70× *g*, 4 °C) for 3 min. The supernatant containing non-parenchymal cells was then transferred to a new conical tube and filled with HBSS to a final volume of 50 mL. After centrifugation (650× *g*, 4 °C) for 7 min, the cell pellets were re-suspended in 10 mL of HBSS. Percoll gradient solution was prepared by layering 25% Percoll™ solution (20 mL, diluted with HBSS) on 50% Percoll™ solution (20 mL, diluted with HBSS). Then, the cell suspension was carefully overlaid onto 25/50% Percoll gradient solution and centrifuged (1800× *g*, 4 °C) for 15 min. The middle layer containing Kupffer cells was transferred to a new conical tube and filled up to 50 mL with HBSS. After centrifugation (650× *g*, 4 °C) for 7 min, the cell pellets in the precipitates were re-suspended in 5 mL of HBSS. The cell viability of isolated Kupffer cells was more than 87% as estimated by trypan blue staining. Kupffer cell activation was assessed by Section 3.10.

### 3.8. ICP-AES Analysis for Si Quantification

SiO_2_ in biological samples were quantified by measuring total Si amount using ICP-AES. The feces (0.1 g), urine (0.2 mL), or cells (0.1 g) were digested in perfluoroalkoxy microwave digestion vessels using a microwave system (ETHOS EASY, Milestone Srl, Sorisole, Italy) with 70% HNO_3_ (6 mL) and 40% HF (1 mL). The microwave system was set at 1600 W and irradiated for 15, 10, and 30 min to reach 120, 160, and 210 °C, respectively, followed by holding at 210 °C for 1 min and cooling at 25 °C [66]. After digestion, the samples were diluted to appropriate volumes with DDW and analyzed by ICP-AES at a wavelength of 212.412 nm with radiofrequency power of 1000 W and plasma gas flow of 12 L/min. The quantitative analytical method was validated by evaluating the linearity (R^2^), recovery (%), CV, RE, LOD, and LOQ. The LOD and LOQ were calculated by the following equations: LOD = 3.3 × σ/S; and LOQ = 10 × σ/S (σ: standard deviation of the response; S: slope of the calibration curve).

### 3.9. Cytokine Release

In vivo cytokine release caused by repeated oral administration of SiO_2_ for 28 d was evaluated using ELISA kits. Plasma samples were obtained by the centrifugation (3000× *g*, 4 °C, 15 min) of the blood at designated time points (0, 1, 14, 28, and 29 d) after oral administration of SiO_2_. The levels of pro-inflammatory (IL-1β, IL-8, and TNF-α) and anti-inflammatory cytokines (IL-10 and TGF-β) were determined by ELISA according to the manufacturer’s protocol.

### 3.10. Flow Cytometric Analysis

In vivo macrophage polarization caused by repeated oral administration SiO_2_ for 28 d was evaluated by flow cytometric analysis. The Kupffer cells (1 × 10^6^ cells in 100 μL HBSS) isolated from the rat liver were washed with staining buffer (1 mL, BD Biosciences), and Fc receptors were pre-blocked with anti-rat CD32 antibody for 15 min on ice. The staining buffer (1 mL) was added to the cells and centrifuged (300× *g*, 4 °C) for 5 min to remove excess antibody. The Kupffer cells were then stained with 100 μL of PE-conjugated anti-rat CD86 antibody (1:200 dilution), M1 surface macrophage marker, and Alexa Fluor 647-conjugated anti-rat CD163 antibody (1:100 dilution), M2 surface macrophage marker, for 30 min on ice in the dark, respectively, and washed with staining buffer (1 mL) repeatedly. Then, the cells were fixed and permeabilized using 250 μL of fixation/permeabilization solution (BD Biosciences) for 20 min on ice in the dark and washed twice with 1 mL of permeabilization/washing buffer (BD Biosciences). The cells were stained with FITC-conjugated anti-rat CD68 antibodies (1:100 dilution) and intracellular pan-macrophage marker for 30 min on ice in the dark, washed with permeabilization/washing buffer (1 mL) twice, and re-suspended in 300 μL of staining buffer. The polarization of Kupffer cells was analyzed with flow cytometer (FACS Canto II, BD Biosciences) by counting 50,000 cells using FlowJo software Ver. 0.8.1 (FlowJo, BD Biosciences).

### 3.11. Cell Culture and Cellular Uptake

The mouse macrophage Raw 264.7 cell line was obtained from Korea Cell Line Bank (Seoul, Republic of Korea) and cultured in RPMI 1640 medium containing 10% FBS, 100 units/mL of penicillin, and 100 μg/mL of streptomycin under 5% CO_2_ atmosphere at 37 °C.

Raw 264.7 cells (1 × 10^6^ cells/6 well) were incubated with SiO_2_ (1000 μg/mL) for 24 h. After washing three times with DPBS, the cells were harvested with a scraper. Then, the cells were centrifuged (850× *g*, 4 °C) for 1 min and re-suspended in 1 mL of DDW. For quantitative analysis of the cellular uptake amount of SiO_2_, the cell suspension was pre-digested with HNO_3_ and HF in a microwave digestion system, and intracellular uptake of SiO_2_ was quantified by ICP-AES analysis, as described in Section 3.8.

### 3.12. Intracellular Fate Determination

To determine intracellular fates of SiO_2_, the cell suspension (1 × 10^6^ cells in 1 mL) was sonicated four times on ice for 10 s at 150 W using an ultrasonic homogenizer (Sonics & Materials Inc., Newtown, CT, USA). Cell suspension without SiO_2_ treatment was used as a control. Half of the cell suspension was subjected to a CPE procedure, and the other half of the suspension was used for ortho-silicic acid analysis. For the CPE procedure, 0.5 mL of the cell suspension was diluted to 7 mL with DDW and the CPE method was applied as previously described [10]. Briefly, 0.5% (*w*/*v*) TX-114 (0.5 mL) and 0.2 M NaCl (0.75 mL) were added to the diluted cell suspension after pH adjustment to 3.0 with HNO_3_. Then, the mixture was diluted to 10 mL with DDW, followed by incubation for 30 min at 45 °C and centrifugation (2500× *g*, 25 °C) for 5 min. The supernatants of the aqueous phase and the precipitates of the TX-114-rich phase were separately collected to quantify Si ions and SiO_2_ particles, respectively. The samples were pre-digested and quantified as described in Section 3.8.

The remaining cell suspension (0.5 mL) was centrifuged (16,000× *g*, 25 °C) for 30 min and the supernatant was used to quantify silicic acid using the molybdenum blue assay, as described in Section 3.5. To prepare the standard solution of ortho-silicic acid in cell suspension, ortho-silicic acid stock solution (1000 μg/mL, adjusted to pH 7 with HCl) was added to the non-treated cell suspension at designated concentrations (0, 20, 40, 60, 80, and 100 μg/mL).

### 3.13. Statistical Analysis

The results were presented as means ± standard deviations. One-way analysis of variance with Tukey’s test in SAS Ver. 9.4 (SAS Institute Inc., Cary, NC, USA) was conducted to determine the significant differences among groups. Statistical significance was accepted for *p*-values of <0.05.

## 4. Conclusions

Oral excretion kinetics and excretion fates of food-additive SiO_2_ particles, fumed SiO_2_ and precipitated SiO_2_, were evaluated following repeated administration for 28 d. Additionally, their effect on in vivo macrophage activation was assessed. Its intracellular fates were further investigated by determining particle, ionic, and bioavailable silicic acid forms inside macrophage Raw 264.7 cells. The results showed that the majority of orally administered SiO_2_ particles were excreted through feces, and that only a small portion of them were eliminated via urine, regardless of manufacturing method. A fecal excreted fate of SiO_2_ occurred predominantly in intact particle form without decomposition, while its primary urinary excreted fate was in the form of bioavailable silicic acid. A delayed excretion of SiO_2_ was observed due to a high dose (2000 mg/kg) during repeated administration period, but returned to normal basal level at 1 d of recovery period, suggesting that SiO_2_ can be eliminated within 24 h at actual usage levels. SiO_2_ particles were mainly present in particle forms, with slightly decomposition into Si ions or conversion into bioavailable silicic acid inside Raw 264.7 cells, implying a mild decomposition and bioconversion of SiO_2_ after cellular uptake. However, no monocyte uptake, Kupffer cells activation, or cytokine release were detected following repeated oral administration of SiO_2_ particles, suggesting their safety. These findings offer valuable insights into the oral toxicokinetics and potential toxicity of food-additive SiO_2_.

## Figures and Tables

**Table 1:** Validation parameters of quantitative analytical method for fumed SiO_2_ and precipitated SiO_2_ (number of repetitions = 5).

Samples		Validation Parameters	Concentrations (μg/mL)			
			10	20	50	100
Feces	Fumed SiO_2_	Recovery (%)	106.39 ± 5.69	92.20 ± 2.36	92.95 ± 0.74	96.27 ± 4.42
		CV (%)	5.35	2.56	0.80	4.59
		RE (%)	6.39	−7.80	−7.05	−3.73
		R^2^	0.9994			
		LOD (μg/g)	185.98			
		LOQ (μg/g)	563.58			
	Precipitated SiO_2_	Recovery (%)	92.49 ± 4.36	91.39 ± 0.70	91.19 ± 2.56	91.05 ± 0.59
		CV (%)	4.72	0.76	2.80	0.65
		RE (%)	−7.51	−8.61	−8.81	−8.95
		R^2^	1.000			
		LOD (μg/g)	165.24			
		LOQ (μg/g)	500.72			
			**Concentrations (μg/mL)**			
			**0.5**	**1**	**5**	**10**
Urine	Fumed SiO_2_	Recovery (%)	97.57 ± 7.77	99.80 ± 4.23	108.21 ± 1.80	105.88 ± 1.99
		CV (%)	7.96	4.23	1.66	1.88
		RE (%)	−2.43	−0.20	8.21	5.88
		R^2^	0.9998			
		LOD (μg/g)	38.17			
		LOQ (μg/g)	115.67			
	Precipitated SiO_2_	Recovery (%)	105.69 ± 7.33	100.55 ± 0.73	96.89 ± 6.70	91.47 ± 1.54
		CV (%)	8.50	0.89	8.47	2.06
		RE (%)	5.69	0.55	−3.11	−8.53
		R^2^	0.9992			
		LOD (μg/g)	24.84			
		LOQ (μg/g)	75.28			

CV: coefficient of variation; RE: relative error; LOD: limit of detection; LOQ: limit of quantification.

## Data Availability

The data presented in this study are available in the article and Supplementary Materials.

## Supplementary Materials

The following are available online at https://www.mdpi.com/article/10.3390/ijms25031614/s1.
