# Supplementary material for: Oral Excretion Kinetics of Food-Additive Silicon Dioxides and Their Effect on In Vivo Macrophage Activation
Source: Int J Mol Sci. 2024 Jan 28;25(3):1614. doi: 10.3390/ijms25031614 (PMC10855107; doi:10.3390/ijms25031614)
Supplement: Supplementary file 1 [file ijms-25-01614-s001.zip › ijms-2821896-supplementary.pdf]

International Journal of *Molecular Sciences*

MDPI

Article

Oral Excretion Kinetics of Food-Additive Silicon Dioxides and Their Effect on In Vivo Macrophage Activation

Ri-Ye Kwon, Su-Min Youn and Soo-Jin Choi *

Division of Applied Food System, Major of Food Science & Technology, Seoul Women's University, Seoul 01797, Republic of Korea; mystic2121@swu.ac.kr (R.-Y.K.); smyoun@swu.ac.kr (S.-M.Y.)

* Correspondence: sjchoi@swu.ac.kr; Tel.: +82-2-970-5634; Fax: +82-2-970-5977

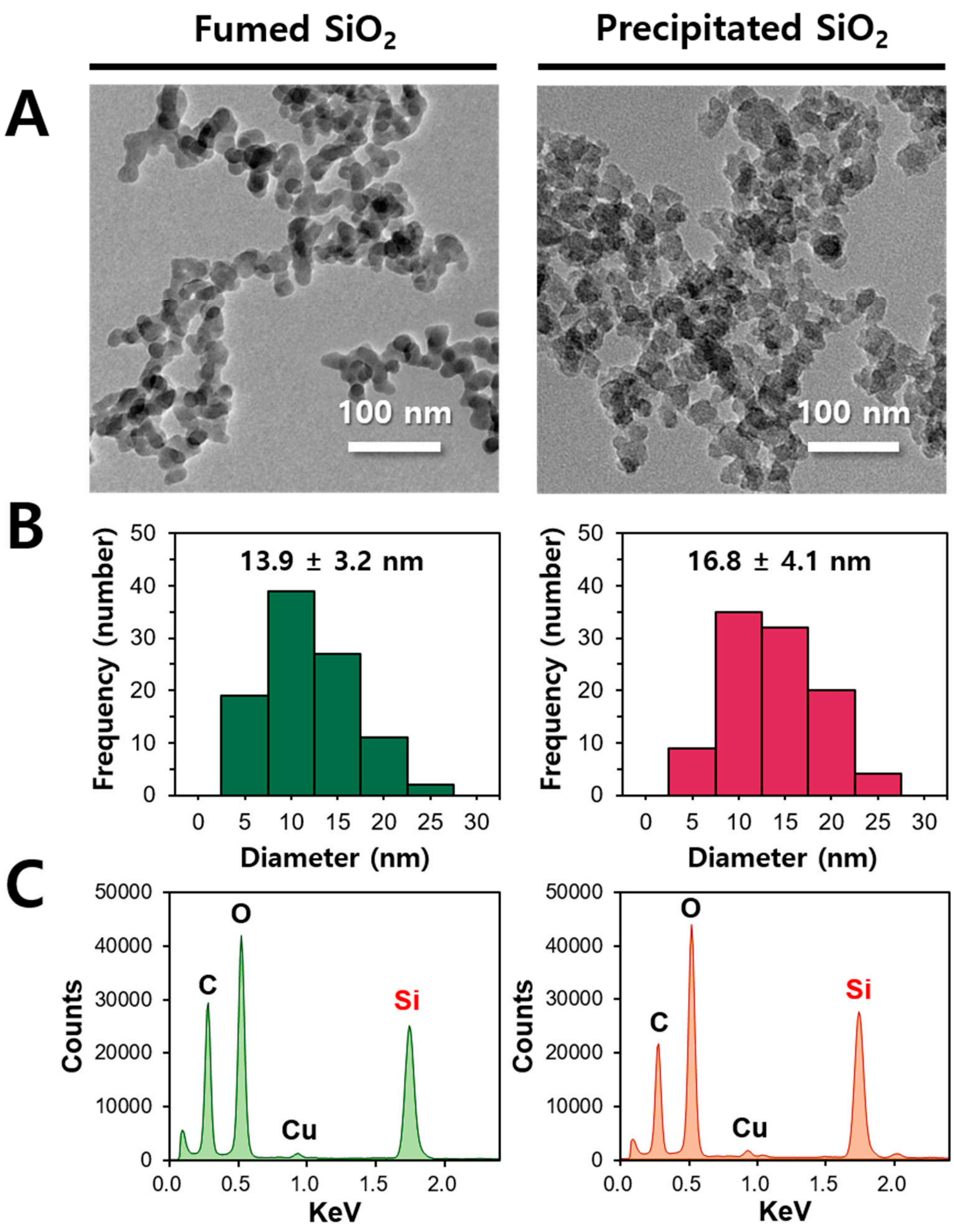

Figure S1. **(A)** Transmission electron microscopy (TEM) images, **(B)** size distribution, and (C) energy dispersive X-ray spectroscopy (EDS) of fumed and precipitated SiO_2. Size distributions and average particle sizes were obtained by randomly selecting 100 parti-cles from the TEM images. No significant differences between fumed SiO_2 and precipitated SiO_2 were found ($P > 0.05$).
